# Global genetic diversity of *var2csa* in *Plasmodium falciparum* with implications for malaria in pregnancy and vaccine development

**DOI:** 10.1038/s41598-018-33767-3

**Published:** 2018-10-18

**Authors:** Ernest Diez Benavente, Damilola R. Oresegun, Paola Florez de Sessions, Eloise M. Walker, Cally Roper, Jamille G. Dombrowski, Rodrigo M. de Souza, Claudio R. F. Marinho, Colin J. Sutherland, Martin L. Hibberd, Fady Mohareb, David A. Baker, Taane G. Clark, Susana Campino

**Affiliations:** 1 Faculty of Infectious and Tropical Diseases, London School of Hygiene & Tropical Medicine, London, United Kingdom; 2 School of Water, Energy and Environment, Applied Bioinformatics, Cranfield University, Cranfield, United Kingdom; 3 Genomics Institute of Singapore, Biopolis, Singapore; 4 Department of Parasitology, Institute of Biomedical Sciences, University of São Paulo, São Paulo, Brazil; 5 Multidisciplinary Center, Federal University of Acre, Acre, Brazil; 6 Faculty of Epidemiology and Population Health, London School of Hygiene and Tropical Medicine, London, United Kingdom

## Abstract

Malaria infection during pregnancy, caused by the sequestering of *Plasmodium falciparum* parasites in the placenta, leads to high infant mortality and maternal morbidity. The parasite-placenta adherence mechanism is mediated by the VAR2CSA protein, a target for natural occurring immunity. Currently, vaccine development is based on its ID1-DBL2Xb domain however little is known about the global genetic diversity of the encoding *var2csa* gene, which could influence vaccine efficacy. In a comprehensive analysis of the *var2csa gene* in >2,000 *P. falciparum* field isolates across 23 countries, we found that *var2csa* is duplicated in high prevalence (>25%), African and Oceanian populations harbour a much higher diversity than other regions, and that insertions/deletions are abundant leading to an underestimation of the diversity of the locus. Further, ID1-DBL2Xb haplotypes associated with adverse birth outcomes are present globally, and African-specific haplotypes exist, which should be incorporated into vaccine design.

## Introduction

Malaria infection during pregnancy, caused by *Plasmodium falciparum* parasites, is a major public health burden in tropical areas of Africa and South East Asia, being responsible for substantial maternal and infant morbidity and mortality, including increased adverse outcomes such as miscarriage, maternal anaemia and low birth weight. There is an estimated 200,000 infant and 10,000 maternal deaths per year caused by placental malaria (PM)^1^. The increased susceptibility to *P. falciparum* infection during pregnancy, regardless of previously acquired malaria immunity, has been attributed to the sequestration of infected erythrocytes in the placenta^2,3^. *P. falciparum* blood stage parasites accumulate in the placenta by adhering to chondroitin sulfate A (CSA)^4^. The interaction between infected erythrocytes and the placental syncytium receptor is mediated by the parasite protein VAR2CSA^5^. The extracellular region of this ~350 kDa cysteine–rich transmembrane protein is formed by six Duffy-binding-like (DBL) domains and binds with high specificity to the CSA receptor^6,7^. The protein is encoded by the *var2csa* gene (~10 Kbp in length), which is the most conserved member of the *var* family that encodes the variable antigen *P. falciparum* erythrocyte membrane protein-1 (PfEMP1)^8^. VAR2CSA is preferentially expressed by placental parasites^9^. Primigravid women with a lack of immunity against these subpopulation of parasites are the most affected, but become less susceptible in subsequent pregnancies^5,10,11^.

The full length *var2csa* gene has been identified on chromosome 12, but additional loci have also been found on other chromosomes (e.g. 1, 5–9) both in laboratory and field isolates^12^. However, links to phenotypic advantage have not yet been established^12–14^. Within a single genome, multiple *var2csa* gene copies are not necessarily identical^12^, and a high proportion of parasites from infected pregnant women have been found to possess multi-*var2csa* variants, suggesting that having multiple copies may be advantageous during disease progression^14^. Parasites with multiple *var2csa* copies may persist longer during pregnancy by having an increased capacity for antigenic variation and evasion of the maternal immune response^12,14^.

Intermittent preventive treatment in pregnancy (IPTp) has contributed to a reduction in PM burden^1^, but currently recommended anti-malarial drugs are threatened by high levels of parasite resistance. The development of a placental malaria vaccine is based on naturally occurring immunity, and two VAR2CSA-based candidates are currently in clinical trials^15,16^. Several studies have compared levels of antibody in the sera of primigravid and multigravid women that recognise specific domains of VAR2CSA, including N-terminal VAR2CSA fragments that have high binding affinity for CSA^15–17^. The purpose of any vaccine for PM is to induce immunity in nulligravid women that would confer protection against PM during subsequent infection, and similarly should boost the immunity acquired by multigravid in endemic areas. It remains to be established whether individual VAR2CSA immunogens are able to induce PM protective immunity analogous to that of naturally-acquired immunity.

Studies looking at *var2csa* genetic diversity in field isolates sourced from pregnant infected women, have found that parasites cluster into five different clades based on the CSA-minimal binding site sequence (ID1-DBL2Xb)^18,19^. A clade identified as 3D7-like was associated with the delivery of infants with lower birthweight^18^, suggesting that VAR2CSA diversity affects pathogenicity and, by inference, antigenicity. Thus, the intra- and inter-population genetic diversity of the *var2csa* gene is likely to affect the efficacy of any vaccine developed based on the VAR2CSA protein, highlighting the need for genetic diversity studies^20^. Therefore, to systematically evaluate the magnitude of this variation, we assess the genetic diversity and structure of the *var2csa*, and estimate copy number profiles, across more than 2,000 *P*. *falciparum* field isolates and laboratory strains spanning 23 countries. We find strong evidence that isolates circulating amongst African populations harbour a much higher diversity in the gene compared to other regions, in both nucleotide variants and structural variants, as well as a significantly higher prevalence of parasites encoding two or more different copies. We report for the first time the global distribution of the different ID1-DBL2Xb clades associated with adverse birth outcomes and an unexpectedly high structural variability of the DBL2x domain across the different populations.

## Results

### *Var2csa* gene copy numbers in laboratory strains

We investigated the number of *var2csa* gene copies present in 21 genomes from laboratory cultured strains sequenced using the PacBio RS-II long-read sequencing platform. These were Dd2 (IndoChina); KH01 and KH02 (Cambodia); D10 (Papua New Guinea); T9/96 and K1 (Thailand); 7G8 and IT (Brazil); HB3 (x2) (Honduras); 3D7 and NF54 (Africa); GA01 (Gabon); GB4 (Ghana); GN01 (Guinea); CD01 (Congo); KE01 (Kenya); SD01 (Sudan); TG01 (Togo); SN01 (Senegal) and ML01 (Mali)^21^. For each strain, contigs for the *var2csa* gene were extracted from assemblies of high-quality reads, and aligned to the 3D7 reference, known to have only one copy of *var2csa*, to generate a phylogenetic tree (S1 Fig.). Almost all strains have single copies of the *var2csa* gene, except HB3 (confirming^13^), D10 and KH01, which have 2 copies. The HB3 copies are closely related and were more similar to each other (93% similarity) than the D10 (87% similarity) and KH01 (88%) pairs. Isolates TG01 and ML01 isolates presented with evidence of multiplicity of infection (MOI>1) and the extra *var2csa* gene copies observed are thought to belong to the different clones in the sample. Investigation of the sequences found 3,401 unique mutations, spanning 2,632 polymorphic sites from a total of 8,011 sites (excluding gaps and without missing data). Also, 601 unique InDels were found, of which 439 were overlapping, leaving a set of 162 non-overlapping InDels.

### Global structural analysis of *var2csa* gene extra copies

We sought to determine the number of *var2csa* gene copies present in *P. falciparum* field isolates (n = 3,125; 23 countries; including from the Pf3k project (https://www.malariagen.net/projects/pf3k)) and laboratory strains (n = 5) with Illumina sequencing data in the public domain. The analytical pipeline is summarised in S2 Fig. After quality control filtering, a total of 2,099 (67.2%) field isolates with non-high multiplicity of infection (clonal for >70% of genome), low numbers of heterozygous single nucleotide polymorphism (SNPs) (<0.015% of total SNPs) and high genome-wide coverage (>30-fold) were retained (S3 Fig.). By comparing the coverage of the larger N-terminal of *var2csa* exon 2 to the average read coverage across the rest of the resident chromosome (see S4 Fig.), we confirmed the inferred copy numbers for 5 laboratory strains (HB3 and D10 have 2 copies; 3D7, 7G8 and GB4 have 1 copy). The presence of extra and different *var2csa* gene copies manifests itself in heterozygous or mixed genotype signatures. Therefore we compared the estimated number of *var2csa* copies to the proportion of mixed calls present in the gene. There was a clear increase in heterozygous calls in the gene for the samples with additional copies, and the mean proportion of heterozygous calls for samples presenting 1 copy is close to zero (S5 Fig.). This approach confirmed the presence of similar and different *var2csa* copies in HB3 (93% similarity) and D10 (87%), respectively. In the field isolates (n = 2,099) we found geographical differences in the frequency of extra copies, where Oceania (21/24, 88%) was highest, followed by African populations (West Africa (172/489, 35%); East Africa (120/409, 29%)), and the lowest frequency was in South East Asia (235/1108, 21%) (test of proportions P < 0.001) (Table S1).

### Characterisation of the *var2csa* gene and genetic diversity in field isolates

**Figure 1:**
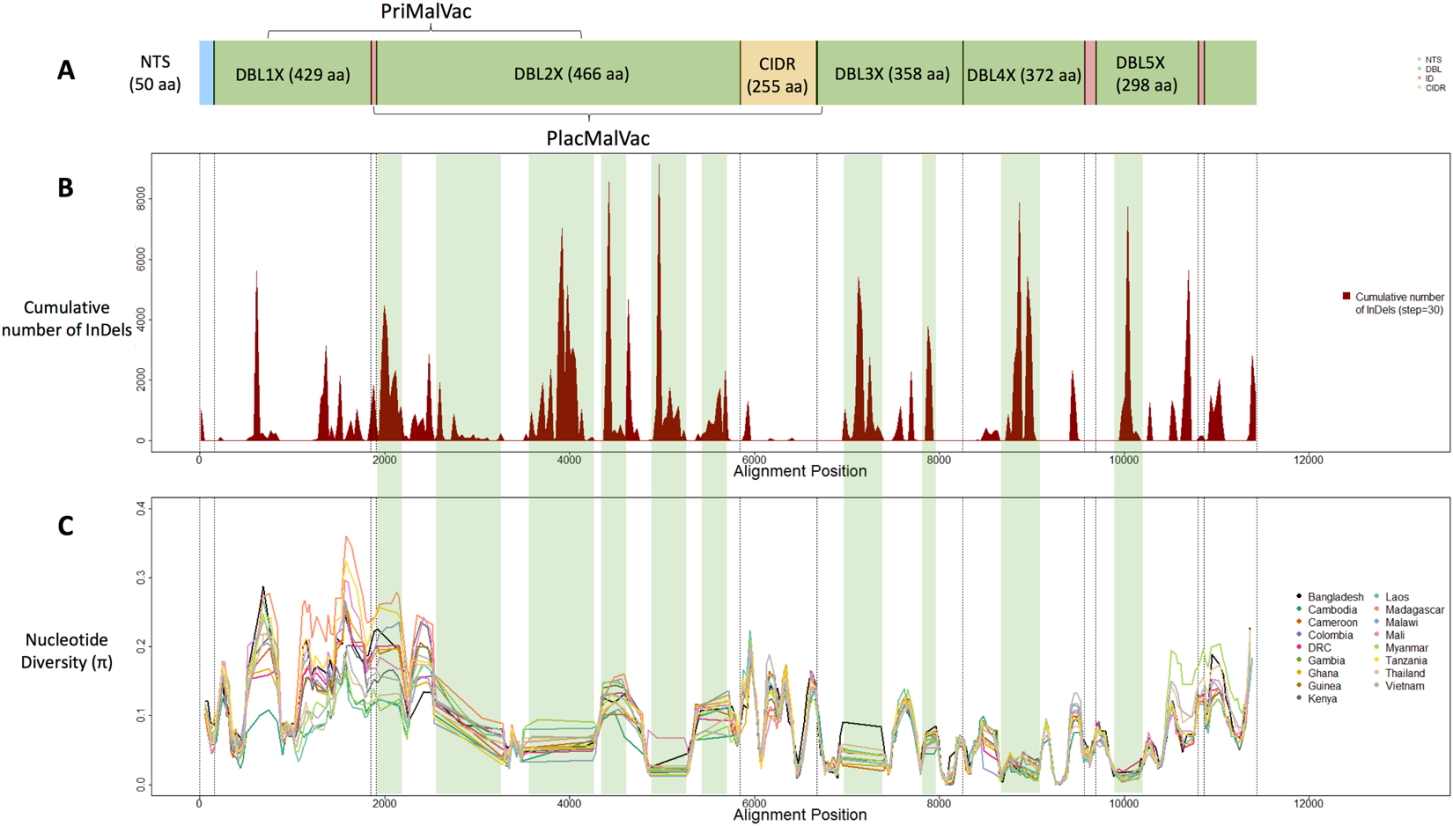
*Plasmodium falciparum var2csa* diversity across a 7 kb region covering the five DBL domains, and 1,249 field isolates. (**A**) Schematic structure of the *var2csa* gene including the N-terminal sequence (NTS, blue), 5 Duffy binding like-Domains (DBL, green), 3 Inter-Domains (ID, red) and the Cysteine-Rich Inter-Domain (CIDR, yellow); the lengths in amino acids of the 3D7 reference are presented in parentheses. (**B**) Accumulation of unique insertions and deletions (InDels) across the *var2csa* gene. (**C**) Distribution of nucleotide diversity (*π*) across the gene and by population. Regions of abnormal (“flat”) nucleotide diversity are highlighted in green (**B** and **C**).

Isolates with a single *var2csa* copy number and low numbers of heterozygous SNPs (<2%) in *var2csa* (S6 Fig.) were identified (n = 1,647), and raw Illumina reads *de-novo* assembled using *velvet* software^22^. A robust multi-software pipeline (see Materials and Methods) led to a high quality multi-alignment of 1,249 sequences, spanning more than 7Kb of the N-terminal end of the *var2csa* gene (Table 1, Fig. 1A and Table S1). The length of non-missing sequence varied per population (median 6,700 bp; inter-quartile range: 6484–6784 bp; Table 1). The assembly pipeline was validated on the GB4, 3D7 and 7G8 Illumina data. When the resulting >7Kb contigs were compared to the Pacbio and capillary sequencing long-read assemblies^23^, there was 100% match for all strains (S7 Fig.).

**Table 1:** Summary of the *Plasmodium falciparum var2csa* assembled 7Kb fragment and its diversity by country using the field isolates with little evidence of mixed infections (n = 1,249).

Population	n	Number sites without missing data	Number of Haplotypes	Haplotype diversity (*Hd*)	Nucleotide diversity (π)	Average no. of nucleotide differences (pairwise comparison)
**Burkina Faso**	**7**	**—**	**—**	**—**	**—**	**—**
**Cameroon**	**49**	**5317**	**44**	**0.995**	**0.0828**	**440**
**Gambia**	**31**	**6700**	**21**	**0.963**	**0.0795**	**533**
**Ghana**	**116**	**6037**	**105**	**0.998**	**0.0798**	**482**
**Guinea**	**33**	**6553**	**32**	**0.998**	**0.0866**	**567**
**Mali**	**15**	**6792**	**15**	**1**	**0.0873**	**593**
**Nigeria**	**3**	**—**	**—**	**—**	**—**	**—**
**DRC**	**77**	**6484**	**71**	**0.998**	**0.0815**	**530**
**Kenya**	**21**	**6784**	**19**	**0.990**	**0.0821**	**557**
**Malawi**	**95**	**5953**	**63**	**0.992**	**0.0819**	**488**
**Tanzania**	**30**	**6578**	**30**	**1**	**0.0892**	**587**
**Uganda**	**4**	**—**	**—**	**—**	**—**	**—**
**Madagascar**	**10**	**6899**	**10**	**1**	**0.0976**	**673**
Bangladesh	11	6830	10	0.982	0.0896	612
*Cambodia*	*360*	*4828*	*46*	*0.911*	*0.0696*	*336*
*Laos*	*40*	*6776*	*29*	*0.979*	*0.0786*	*533*
*Myanmar*	*72*	*6784*	*27*	*0.949*	*0.0836*	*567*
*Thailand*	*184*	*6651*	*47*	*0.968*	*0.0809*	*538*
*Vietnam*	*68*	*6712*	*30*	*0.893*	*0.0794*	*533*
PNG	3	—	—	—	—	—
Brazil	3	—	—	—	—	—
Colombia	10	7045	6	0.889	0.0775	546
Peru	6	—	—	—	—	—

bolded are African countries, italicised are South East Asian countries, DRC = Democratic Republic of Congo; PNG = Papua New Guinea.

Across the 1,249 samples, we identified 1,387 polymorphic SNPs. Haplotype diversity (*Hd)* was high and invariant between the majority of the different Duffy-Binding-Like (DBL) domains (*Hd* = 0.986), consistent with previous work^19^. There was some evidence of higher haplotype diversity in African populations (mean *Hd* = 0.993) compared to South East Asian populations (mean *Hd* = 0.940) (Table 1, T-test P = 0.03). The nucleotide diversity (*π*) was more variable across domains, with higher values towards the N-terminus of the protein (Table 2). The DBL2x region of the CSA minimal binding domain had the lowest nucleotide diversity. The diversity trends observed were consistent across populations (Fig. 1C). Consistent with the haplotype diversity result, the overall nucleotide diversity in African populations (mean π = 0.085) was marginally greater than for South East Asian parasites (mean π = 0.078), but not statistically significant (T-test P = 0.06).

**Table 2:** Diversity statistics across the different domains of the *var2csa* gene in 1,249 isolates of *P. falciparum*.

Domain	Length in amino acids in 3D7 reference	Region in alignment	% of InDel positions	Mean length	Standard Deviation (length)	Positions without missing data	Variant Sites	Variant Sites (%)	No. Haplotypes (n = 1,249)	Haplotype Diversity *(Hd)*	Nucleotide diversity *(π)*
NTS	50	1–153	3	151.2	1.3	132	48	36	285	0.977	0.105
DBL1X	429	154–1852	36	1300.0	7.8	856	391	46	436	0.986	0.115
**ID1**	**14**	**1853–1907**	**98**	**42.6**	**3.9**	**56**	**1**	**2**	**2**	**0.003**	**0.003**
**DBL2X**	**466**	**1908–5844**	**61**	**1428.4**	**39.7**	**657**	**188**	**29**	**437**	**0.986**	**0.071**
**CIDR**	**255**	**5845–6670**	**13**	**769.0**	**1.6**	**636**	**207**	**33**	**436**	**0.986**	**0.103**
DBL3X	358	6671–8253	30	1102.2	21.6	890	172	19	449	0.986	0.044
DBL4X	372	8254–9568	27	1103.9	14.9	808	121	15	450	0.986	0.032
ID2	40	9569–9691	0	122.0	0	122	25	20	133	0.958	0.045
DBL5X	298	9692–10799	32	885.3	37.6	668	202	30	414	0.986	0.063
ID3	20	10800–10863	13	58.8	3.3	56	32	57	56	0.919	0.193

InDel Insertions and deletions; Bolded is the CSA minal binding-domain.

### The presence of insertions and deletions

The *de-novo* assembly of the N-terminal *var2csa* gene fragment, encoding the 5 extra-cellular DBL domains, enabled the study of insertions and deletions (InDels). InDels concentrated around specific regions in the gene (Fig. 1B), where peaks in density coincide with regions of flat nucleotide diversity (highlighted in green in Fig. 1C). The presence of high density, low frequency InDels in the domains leads to an underestimation of both the nucleotide diversity and variation in their length (S8 Fig.). The DBL2X domain has the highest density of InDels **(**Table 2), with sequence lengths across samples (430 to 550 amino acids) twice those of the 3D7 reference, and greater diversity than the other domains. It is unclear how the diversity created by these InDels might affect the structure of the protein and therefore, both its binding affinity to the CSA receptor during pregnancy and recognition by vaccine-generated antibodies. However, in extreme cases the same DBL domain differs by more than 120 amino acids in length.

### Population structure analysis of the ID1-DBL2Xb region

**Figure 2:**
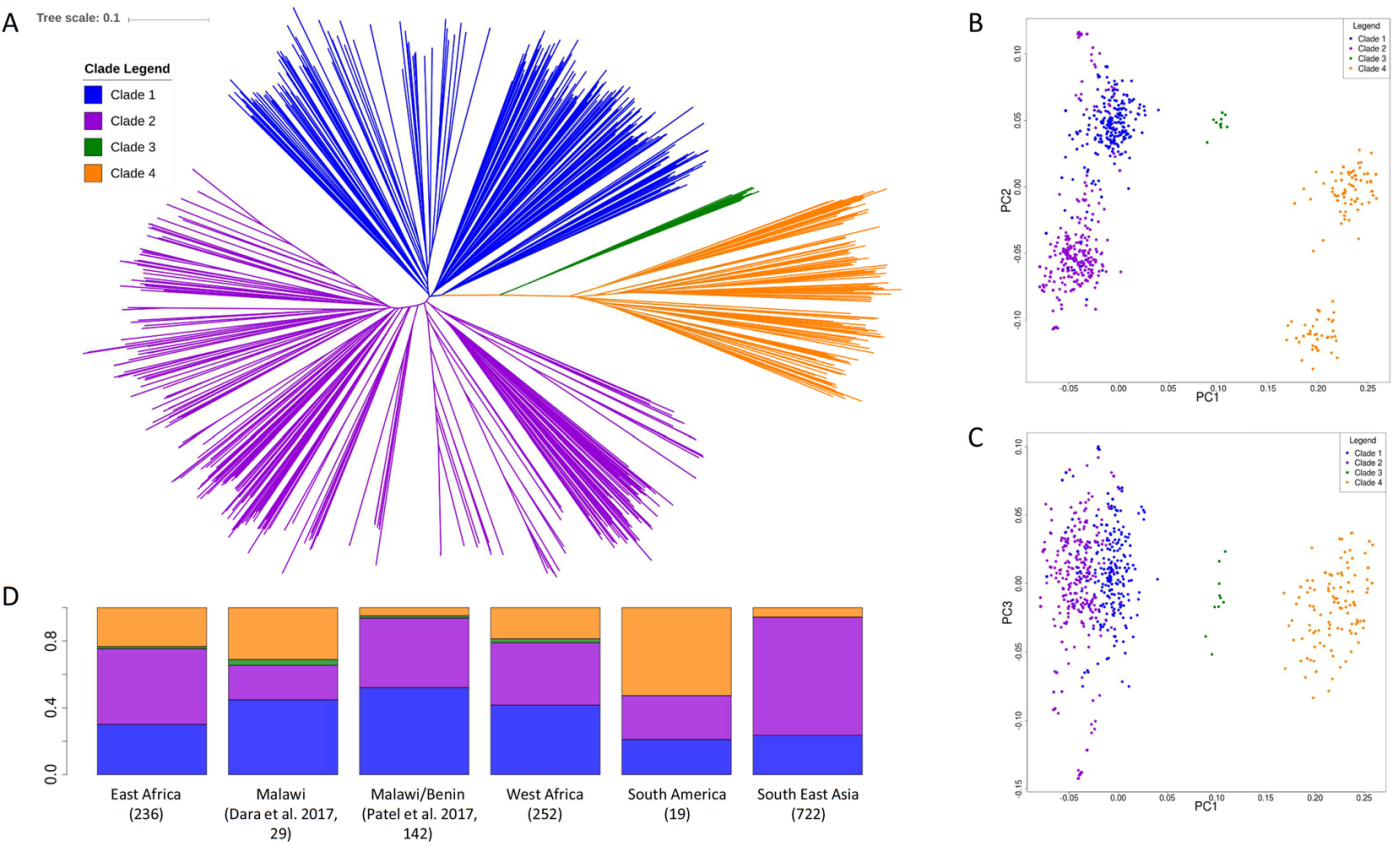
Population structure using the *ID1-DBL2Xb* protein sequences (**A**) Four distinct clades are identified, with some overlap to the clades found in^18^, where Clade 1 is 3D7-like and Clade 2 is FCR3-like. The PCA analysis (**B** and **C**) supports the separation of these clades and reveals the proximity of Clades 1 and 2. (**D**) The distribution of clades across the different regions and previous studies, with three of the clades present across all the populations (Clades 1, 2 and 4); 3D7-like clade associated with adverse outcome in pregnancy is in West Africa (41.2%), East Africa (27.5%), South East Asia (23.5%) and South America (20%); Clade 3 is present in African parasite populations and Clade 4 is predominantly in African populations.

The ID1-DBL2Xb region is the CSA-minimal binding domain and several studies have aimed to characterize its protein structure^18,19^. By combining the published ID1-DBL2Xb sequences (n = 124) with those extracted from the *de-novo* assembled sequences (n = 1,249), we assessed whether the variants led to clustering of *P. falciparum* parasites (Neighbour-Joining tree (Fig. 2A); principal component analysis (Fig. 2B,C)). Four clades were observed, where Clades 1 and 2 have been previously identified as the 3D7-like and FCR3-like^18^, respectively (Fig. 2D, Table S2). All geographical regions were represented in Clades 1, 2 and 4, but Clade 3 contains only parasites from Africa (S9 Fig.). The presence of Clade 1 (3D7-like) has been associated with low birthweight in African populations^18^, and had highest representation in West African populations (41.7%) (East Africa 27.5%; South East Asia 23.5%). A rarefaction curve analysis of the haplotype diversity in the ID1-DBL2Xb region **(**S10A,B Fig.) revealed much greater diversity and more unique haplotypes in African parasites when compared to South East Asian populations. This observation is confirmed by the neighbourhood joining tree analysis (S10C–E Fig.), where the individual African populations have much longer distance at the tips than the South East Asian populations.

### The open reading frame (ORF) element upstream of the *var2csa* gene is highly conserved

The ORF region of the *var2csa* plays a role in regulating expression of the gene^24^. Analysis of the complete sequences (n = 1,249; S2 Data) revealed that these elements harbour little diversity and are highly conserved across geographical regions and populations (mean π = 0.02) (Table S3). The clustering of *var2csa* genes into four clades is not complemented by a similar pattern in the ORF regulatory region, which seems to indicate that the gene expression regulatory function of this region might be conserved across clades.

## Discussion

The VAR2CSA protein is a vaccine candidate for PM^25^ and is the basis of two vaccines currently in Phase I clinical trials^15,16^. It is therefore essential to understand the genetic and structural diversity of *var2csa* in natural populations of *P. falciparum* parasites, to predict the impact of potential vaccines, and improve the understanding of the mechanisms by which the malaria parasites sequester in the placenta. It has been suggested that parasite populations with multiple copies of the *var2csa* gene persist longer during pregnancy, hypothesising that this could be due to the ability of these parasites to generate a wider diversity of antigenic variation^14^. Across global field samples (n = 2,099), all populations had evidence of extra copies (27% on average, broadly consistent with^14^), but the prevalence was higher in African and Papua New Guinean parasites. For the African populations this could be due to the higher immune pressure that this gene might be under in higher transmission settings. Whilst, for PNG it could be due to a founder effect arising from the parasite population’s mixed Asian and African ancestry^26^. It is possible the degree of copy number variation in the population-based field isolates sourced predominantly from children with malaria could be different to that found in pregnant mothers. However, the *var2csa* sequences from pregnant mothers included in our analysis overlapped with the sequences and resulting tree clusters observed in the field isolates.

By applying a pipeline validated using Pacbio sequenced strains, 7 kb fragments encoding the VAR2CSA extra-cellular domains were assembled across 1,249 field isolates with little evidence of mixed infections. The diversity was highest towards the N-terminus of the protein, as seen previously^19^ and, in general, higher in African parasite populations compared to South East Asian ones. This result is consistent with African genomes being older and with the higher transmission rates in that continent currently. InDels have been a neglected source of variation and diversity in *var2csa* studies. The presence of a high number of low frequency InDels, which had the highest density in regions with flat nucleotide diversity, revealed that the level of diversity was underestimated. Comparing across the 5 VAR2CSA domains, the highest density of InDels was found in the DBL2X (part of CSA minimal binding domain), leading to the greatest variability in sequence length, where up to an additional 120 amino acid insertions were present. The impact of small in-frame InDels and short frame shifts on the protein structure of the DBL domain and their binding capabilities are not clear, and structural modelling approaches are difficult to scale-up to the levels of variation observed. A large number of the SNPs and InDels could lead to important changes in the amino acid sequence while conserving the overall gene structure. However, this should be fully explored to understand any impact on antibody binding affinity and CSA-binding in placental malaria. Further phenotypic characterization is required to evaluate the contribution of the diversity observed in the gene at both SNP and InDel level to parasite sequestration in the placenta, which could provide insight into the mechanisms by which the infection causes the associated adverse outcomes during pregnancy.

A recent study in pregnant women in Malawi and Benin identified 5 clades of the ID1-DBL2Xb domain of the *var2csa* gene and found an association between the infection of parasites harbouring the 3D7-like sequence and low birthweight^18^. Our work suggests that there are four main ID1-DBL2Xb domain clades, including a 3D7-like (Clade 1) and FCR3-like (Clade 2). Two of the previously reported clades appear to be too homogenous to be separated in our much larger dataset. Three of the four remaining clades (1, 2 and 4) were present across all the regions, including Clade 1, which was found in West Africa (41.2%), East Africa (27.5%), South East Asia (23.5%) and South America (21.1%), consistent with previous findings in Malawi (44.8%)^19^ and Malawi and Benin (52.1%)^18^. Clade 3 isolates appear to be rare (<1% overall) and almost exclusively present in African parasites. Previous studies^18^ have focused on the effect of the major clades have on pregnancy outcomes, but these data highlight the presence of Clade 4 isolates across very diverse regions very diverse regions globally, therefore stressing the need to further investigate the relationship between the prevalence of the different parasite clades and the pregnancy outcomes. Countries outside of sub-Saharan Africa such as the South East Asian region where the Clade 1 (3D7-like) has been reported for the first time, are of particular interest given that there is no evidence on the impact that parasites harbouring Clade 1 VAR2CSA protein have on pregnancy in these host populations. Furthermore, studies of the potential impact of the less prevalent Clades 3 and 4 on adverse outcomes in pregnancy are needed.

An analysis of the ID1-DBL2Xb domain haplotypes revealed higher diversity across African populations compared to South East Asian populations. A higher proportion of unique haplotypes in African parasites is likely to be a reflection of the higher transmission intensity. These diversity patterns suggest that introduction of a vaccine based on one single haplotype or a few heterologous haplotypes might be of greater benefit in South East Asian populations, although it is in Africa that the vaccine is most needed. Our work suggests that it would be advisable to consider the four clades of related *var2csa* haplotypes when testing the efficacy of the vaccine and, if possible, including heterologous haplotypes for the African specific clades found in this study in future vaccine design.

Overall, our study reveals the genetic diversity of the *var2csa* gene across more than 23 countries, demonstrating that SNPs and InDels are frequent at this locus, and generate considerable haplotype diversity, especially in African parasites. We also report a high frequency of multiple *var2csa* gene copies in the genome of field isolates. Further molecular and association studies are essential to understand the effect of VAR2CSA variants and extra gene copies on parasite sequestration in the placenta, the outcome of pregnancy and vaccine efficacy. Our findings can be used to support the assessment and development of new preventive tools against placental malaria, including the design of new vaccines that are robust to regional genetic diversity.

## Materials and Methods

### Samples, sequence data and processing

High-quality and high molecular weight DNA (20 µg) was purified from laboratory strains (D10, K1, HB3, T9/96 and NF54) using the Qiagen Genomic tip 20/G. Sequencing data for D10 (Papua New Guinea), T9/96 (Thailand), HB3 (Honduras), K1 (Thailand), NF54 (3D7 Parental line) strains were generated on Pacific Biosciences (PacBio) RS-II long read technology at the Genome Institute Singapore and were complemented by similar data for 7G8 and IT (Brazil), GB4 (Ghana), GN01 (Guinea), CD01 (Congo), Dd2 (IndoChina), KE01 (Kenya), KH01 and KH02 (Cambodia), GA01 (Gabon), SD01 (Sudan), TG01 (Togo), SN01 (Senegal) and ML01 (Mali) in the public domain (ftp://ftp.sanger.ac.uk/pub/project/pathogens/Plasmodium/falciparum/PF3K/PilotReferenceGenomes/GenomeSequence/Version1/). High quality sequencing reads (range: 33,855–66,185 reads) were assembled using Hierarchical Genome Assembly Process HGAP3 and corrected using Illumina reads when available, implemented in the SMRT Portal software suite and following previously described methods^27^. Overlaps between the start and end of large contigs were found using *Mummer* software^28^ and removed using in-house scripts.

Raw sequencing data from Illumina data was available for previously published *P. falciparum* strains (3D7, HB3, D10, 7G8 and GB4)^29,30^ and isolates from East Africa (Kenya (n = 38), Malawi (353), Tanzania (63), Uganda (12), Madagascar (18)), West Africa (Burkina Faso (48), Gambia (63), Ghana (443), Guinea (116), Mali (55), Nigeria (6), Cameroon (127)), Central Africa (Democratic Republic of Congo (DRC) (232)), South America (Colombia (15), Peru (9), Brazil (3)), South Asia (Bangladesh (53)) and South East Asia and Oceania (Cambodia (649), Laos (112), Myanmar (134), Papua New Guinea (26), Thailand (326), Vietnam (199))^31^. Public accession numbers for raw sequence data analysed are contained in SRA studies ERP000190 and ERP000199, as well as being accessible from the Pf3k project website (https://www.malariagen.net/projects/pf3k). All Illumina short reads were mapped to the 3D7 reference genome (version 3.0) using *bwa-mem* (version 0.7.17)^32^. SNPs and small InDels were called from the alignment *bam* files using *samtools* and *bcf/vcftools* (version 1.5) with default settings^33^. Only those variants with quality scores in excess of 30 (indicating an error rate less than 1 per 1000 bp) and with minimum coverage of 10 were retained^31^. The pipeline is summarised in S2 Fig. In total, the dataset contains 1,649 isolates and 1,513,940 high quality SNPs; 47.3% within genes and 5.2% have a minor allele frequency greater than 1%. A principal component analysis (not shown) based on pairwise SNP differences between isolates did not reveal any geographic outliers. We used the proportion of heterozygous calls per sample (>0.015%) as well as the fraction of genome indicating Multiplicity Of Infection (MOI)>1 obtained using estMOI (>30%)^34^ as described previously^35^ to remove samples with MOI>1 (S3 Fig.).

### Characterisation of the *var2csa* gene and copy number

The Pacbio sequencing contigs that contained the *var2csa* gene were identified, and mapped to the 3D7 reference genome, allowing the direct assessment of copy numbers. Similarly, the *var2csa* gene was characterised from contigs constructed through *de novo* assembly of Illumina short reads using *velvet software*^22^. The resulting contigs obtained were aligned and assembly errors were corrected manually using *Aliview*^36^. Once corrected the sequences were trimmed from the N-Terminal to form a 7 kb fragment which was then translated using *OrfFinder*^37^ and sequences presenting more than one ORF were excluded from further analysis. The approach was validated by comparing the contigs obtained from PacBio and Illumina platforms, using 3D7, 7G8 and GB4 samples with both sets of data (S8 Fig.). Illumina data were also used to infer copy numbers. In particular, *Delly* software (version 0.7.7)^38^ was used to calculate genomic coverage from the alignment bam files, and the *Control-FREEC tool* (Version 10.6)^39^ was used to estimate copy number based on GC content corrected ratios of coverage using a sliding window of 500 bp and a step size of 100 bp^39^. We also estimated copy number based on the ratio of the average *var2csa* gene coverage against the average gene coverage in the rest of the genome, optimised and tested using the HB3, D10, GB4, 3D7, and 7G8 strains.

### Genetic diversity within and across populations

The selection of the samples for genetic diversity analysis (n = 1,649) focused on those with the presence of a single copy of the *var2csa* gene based on coverage (S4 Fig.) and a maximum of 2% of bases in the *var2csa* gene presenting heterozygous calls in the gene (S7 Fig.). We then screened the contigs assembled using Blast alignment^37^ to the 3D7 reference of the *var2csa* gene and retrieved 1,317 isolate sequences with a hit that contained a contig of more than 8 kb in length. We aligned the sequences using *Mafft*^40^ with default parameters, reverse-complemented when needed and extracted the region corresponding to the *var2csa* gene and µORF region using *AliView*^36^. The alignment was manually curated to remove poly-Ns added by the assembly software as well as obvious assembly errors. The final set of sequences was then translated using *orfinder*^37^ and a further 72 sequences presenting fragmented translation were excluded. The final dataset consisted of 1,249 sequences spanning 7Kb of the N-terminal end of the *var2csa* gene (Table 1, S1 Data).

The *DnaSP* (version 5)^41^ and the *ape* and *agenet*^42^ R libraries were used to compute population genetic parameters at the region and country levels, including nucleotide diversity (π), the average number of nucleotide differences per site between any two given sequences and the haplotype diversity (Hd), which is the probability that two randomly sampled haplotypes are different. We also calculated distance matrices using R package *seqinr*^43^ in order to generate a Principal Component Analysis (PCA) of the nucleotide and protein sequences and the corresponding Neighbour-Joining trees. A *k-means* approach was used in R to obtain the clusters observed in the neighbour joining trees. The *vegan* R package was used to calculate the rarefaction curves using the ID1-DBL2Xb region haplotypes by region and country.

## Electronic supplementary material

Supplementary Information

## Data Availability

Public accession numbers for raw sequence data analysed are contained in SRA studies ERP000190 and ERP000199, as well as being accessible from the Pf3k project website (https://www.malariagen.net/projects/pf3k). Data was complemented by PacBio data in the public domain (ftp://ftp.sanger.ac.uk/pub/project/pathogens/Plasmodium/falciparum/PF3K/PilotReferenceGenomes/GenomeSequence/Version1/).
