# Supplementary material for: Global genetic diversity of var2csa in Plasmodium falciparum with implications for malaria in pregnancy and vaccine development
Source: Sci Rep. 2018 Oct 18;8:15429. doi: 10.1038/s41598-018-33767-3 (PMC6193930; doi:10.1038/s41598-018-33767-3)
Supplement: Supplementary file 1 — Supplementary Information [file 41598_2018_33767_MOESM1_ESM.pdf]

Global genetic diversity of *var2csa* in *Plasmodium falciparum* with implications for malaria in pregnancy and vaccine development

Ernest Diez Benavente

Damilola R Oresegun

Paola Florez de Sessions

Eloise M. Walker

Cally Roper

Jamille G. Dombrowski

Rodrigo M. Souza

Claudio R. F. Marinho

Colin J. Sutherland

Martin L Hibberd

Fady Mohareb

David A. Baker

Taane G. Clark

Susana Campino

S1 figure

Phylogenetic tree for *P. falciparum* laboratory strains constructed using the *var2csa* alignments reveals two near identical gene copies for HB3 and distinct copies for D10 and KH01. Two sequences of each HB3 copy were obtained from two different PacBio runs and were found to be identical, highlighting the robustness of the approach. No significant clustering by geography was found for other samples, as expected from the patterns shown by the short read data.

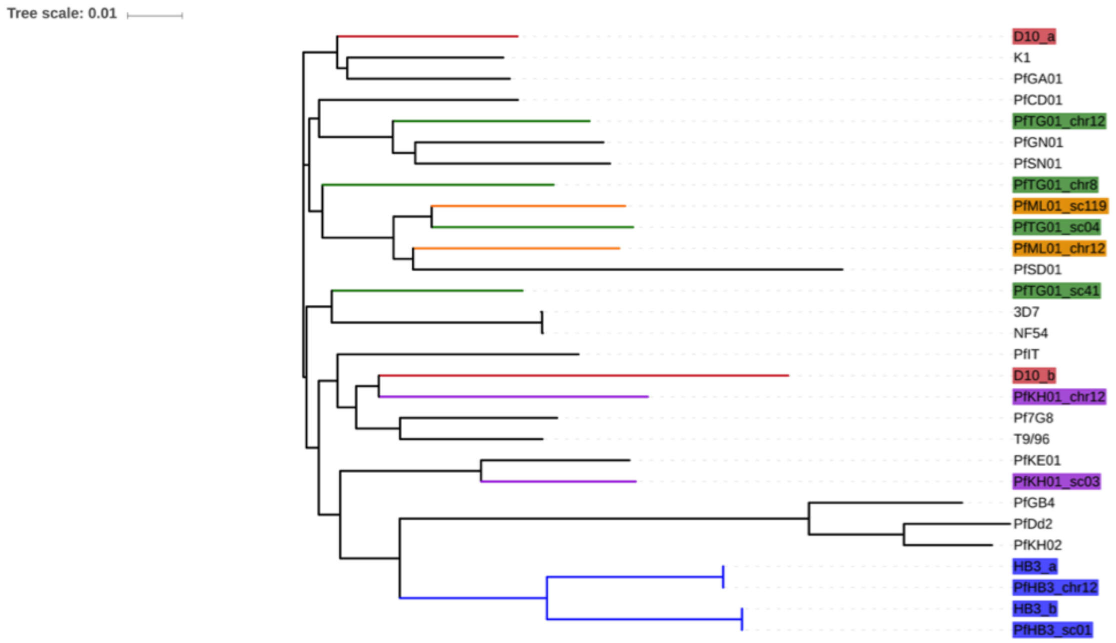

D10 (Papua New Guinea), T9/96 (Thailand), HB3 (Honduras), K1 (Thailand), and NF54 (3D7 Parental line), GN01 (Guinea), CD01 (Congo), Dd2 (IndoChina), KE01 (Kenya), KH01 and KH02 (Cambodia), GA01 (Gabon), IT (Brazil), SD01 (Sudan), TG01 (Togo), SN01 (Senegal) and ML01 (Mali); samples with extra copies are colour coded.

S2 figure

Summary of the analytical and bioinformatics pipelines used

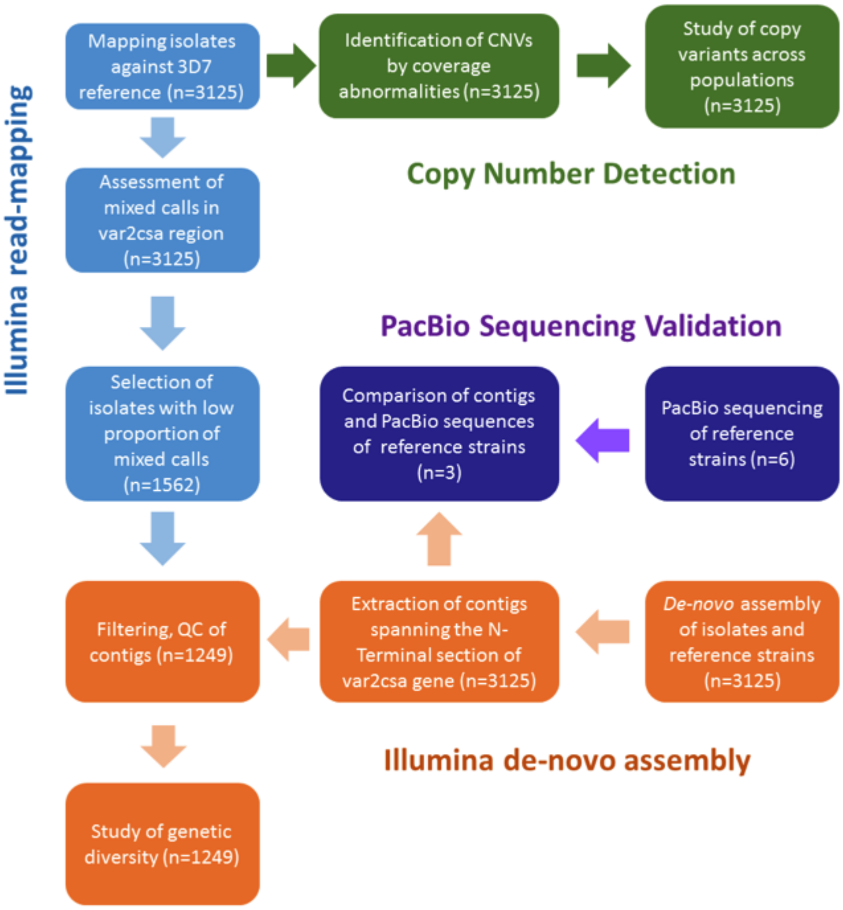

S3 figure

The filtered *P. falciparum* isolates based on MOI and heterozygous calls* (n=2,099; with >70% of their genome with multiplicity of infection of 1 and <1.5% of heterozygous SNP calls)

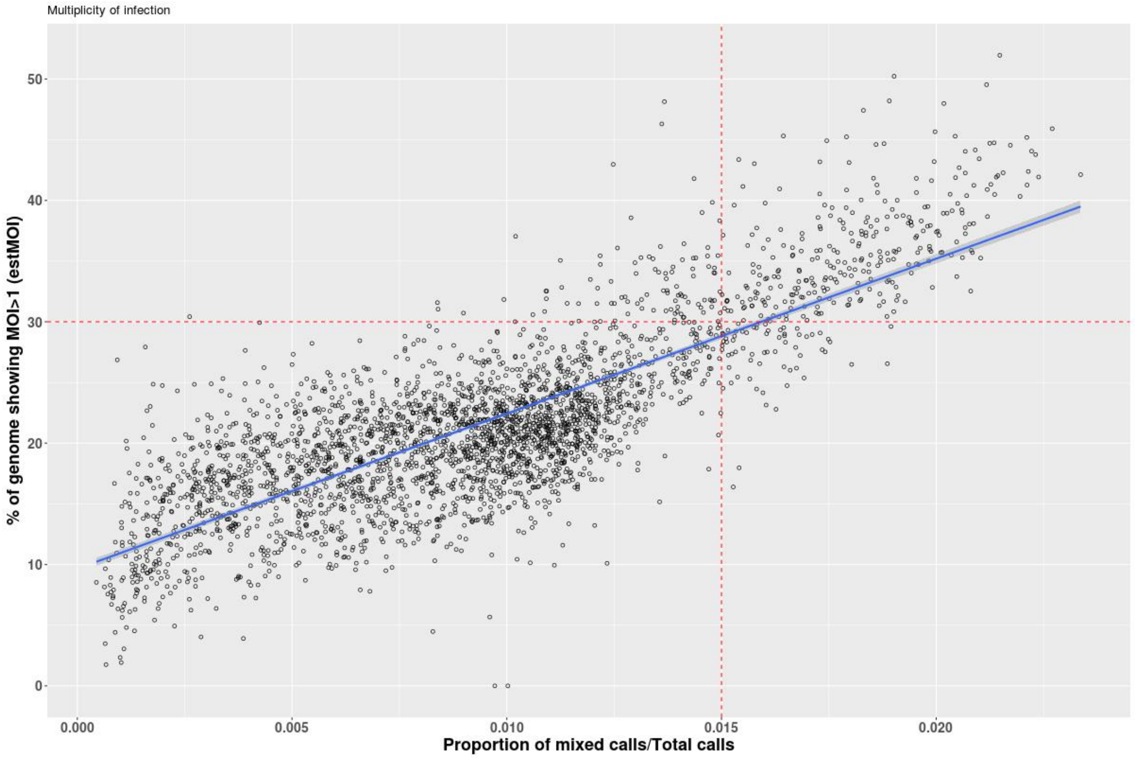

*All samples have >30-fold genome-wide coverage

S4 figure

Estimating copy number in the *var2csa* gene using coverage in 4 *P. falciparum* laboratory strains

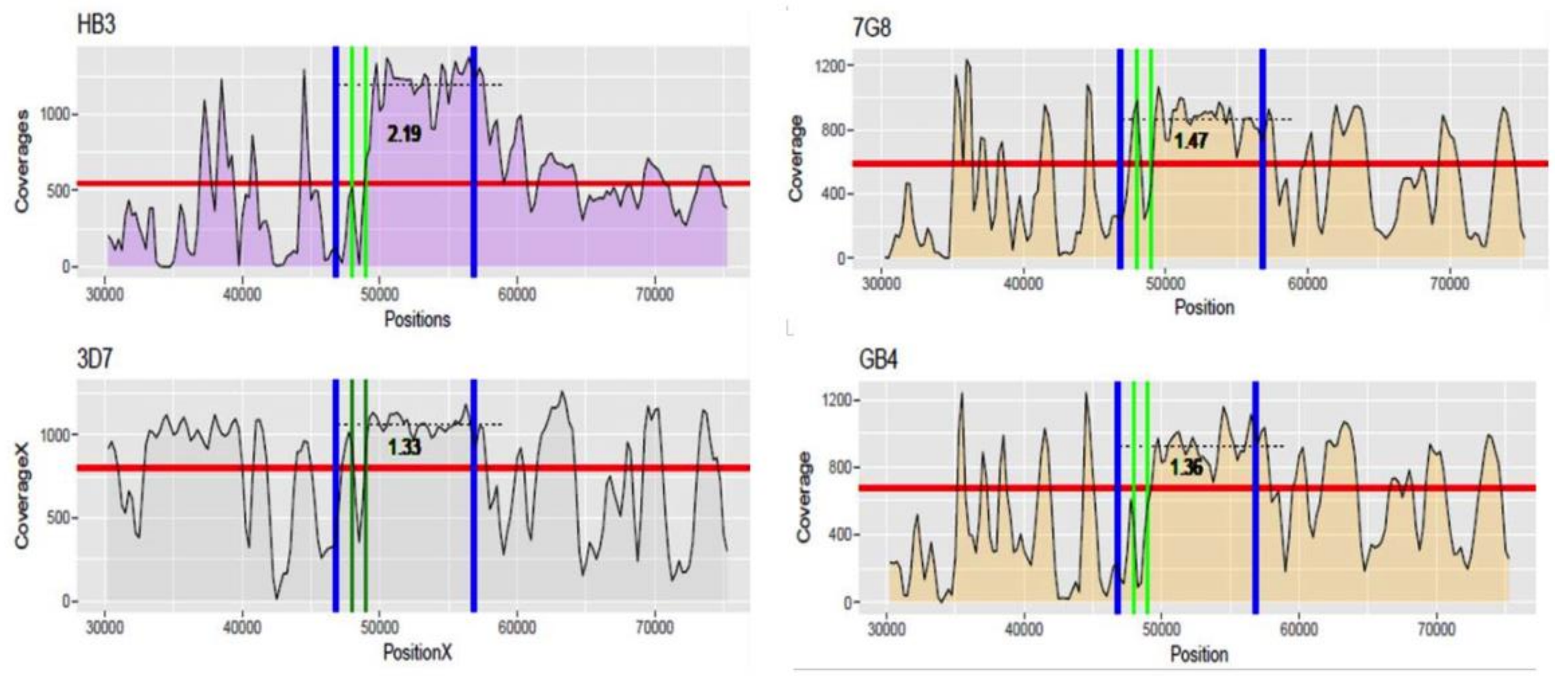

S5 figure

***Var2csa* copy number distributions in *P. falciparum* and mixed SNP calls across the samples, including 5 laboratory strains with Illumina data available**

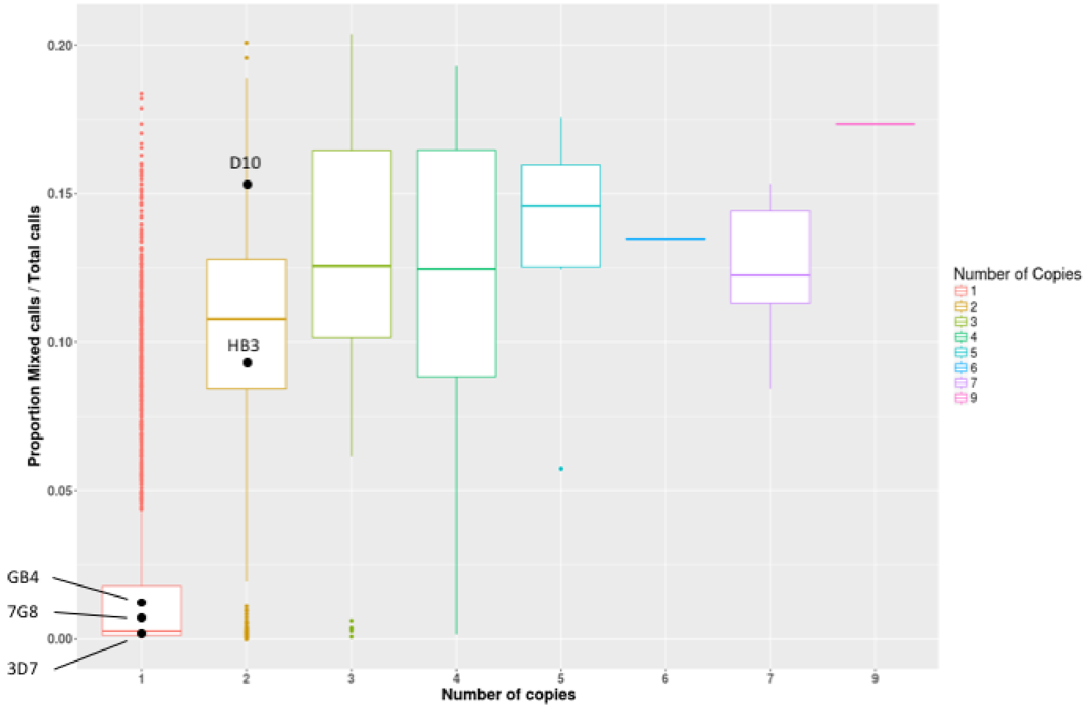

S6 figure

The multiplicity of infection (MOI*) [32] in the genome and the proportion of mixed calls in the *var2csa* gene in *P. falciparum*

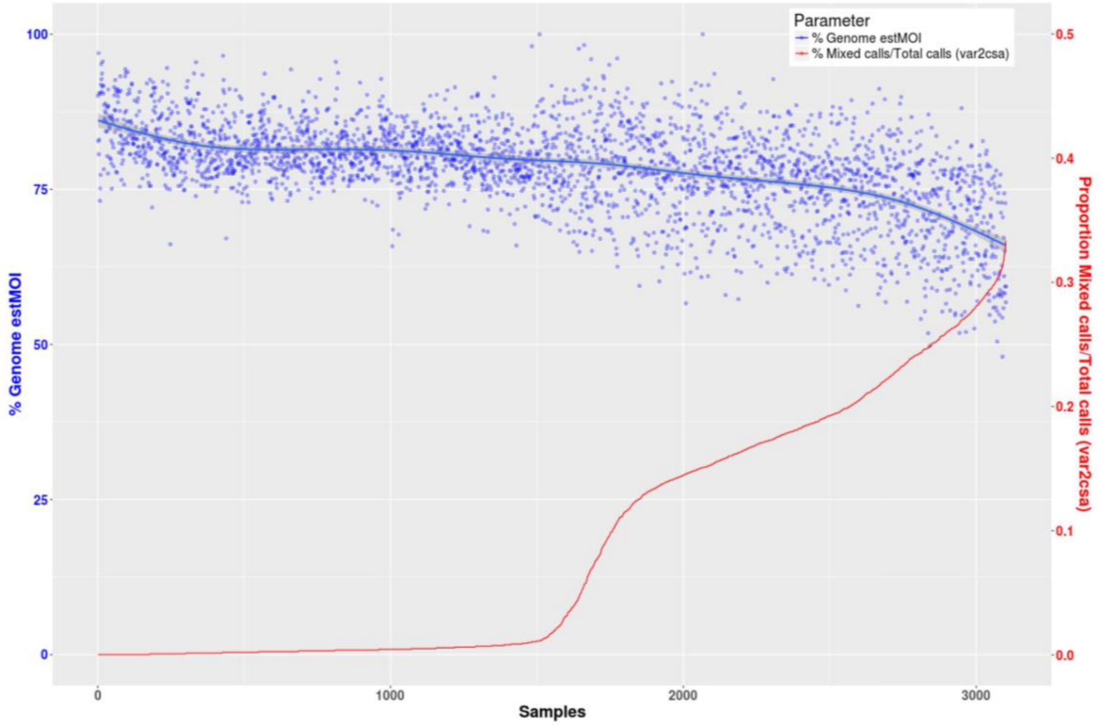

S7 figure

Near perfect matching of the assembled *P. falciparum* strain *var2csa* sequences from our pipeline compared to the long-read gold standard

3D7 Illumina assembled

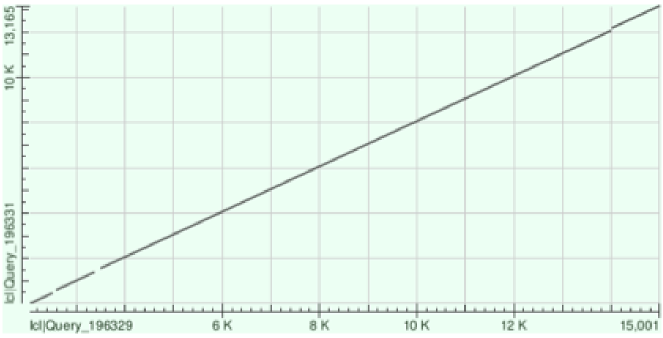

3D7 var2csa reference strain (100% match)

GB4 Illumina assembled

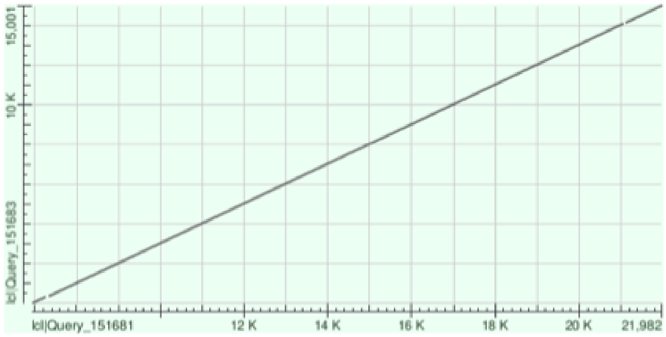

GB4 long-read assembled (100% match)

7G8 Illumina assembled

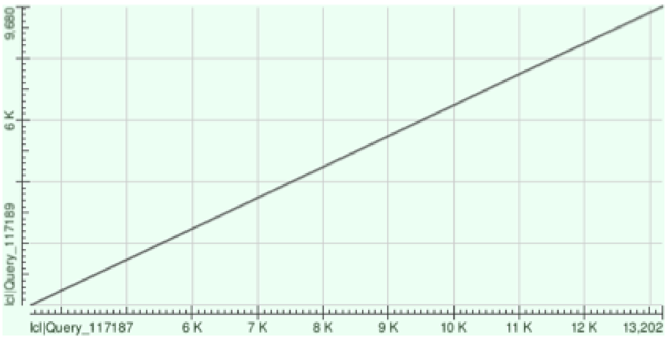

7G8 long-read assembled (100% match)

S8 figure

Distribution of the lengths of the different DBL domains in the *var2csa* gene of *P. falciparum* across the field samples reveals increased length distribution for DBL2X domain (n=1,249)

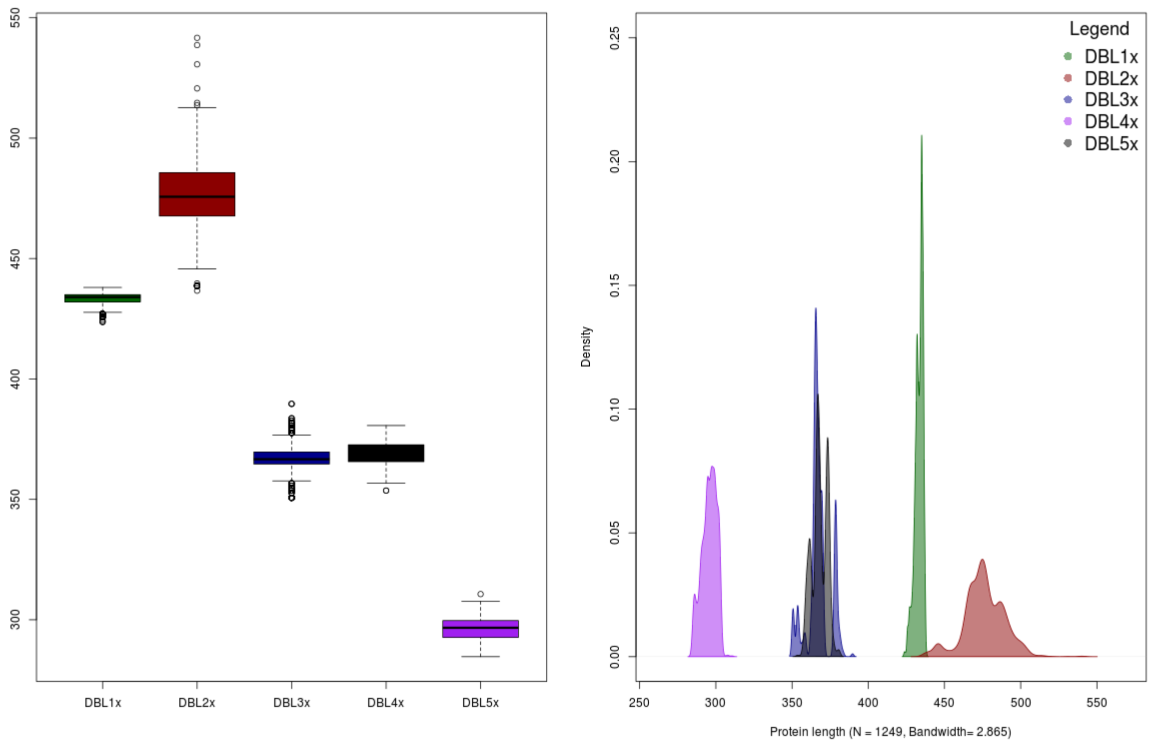

S9 figure

Global map of the distribution of the four clades based on the ID1-DBL2Xb region of the *var2csa* gene in *Plasmodium falciparum*

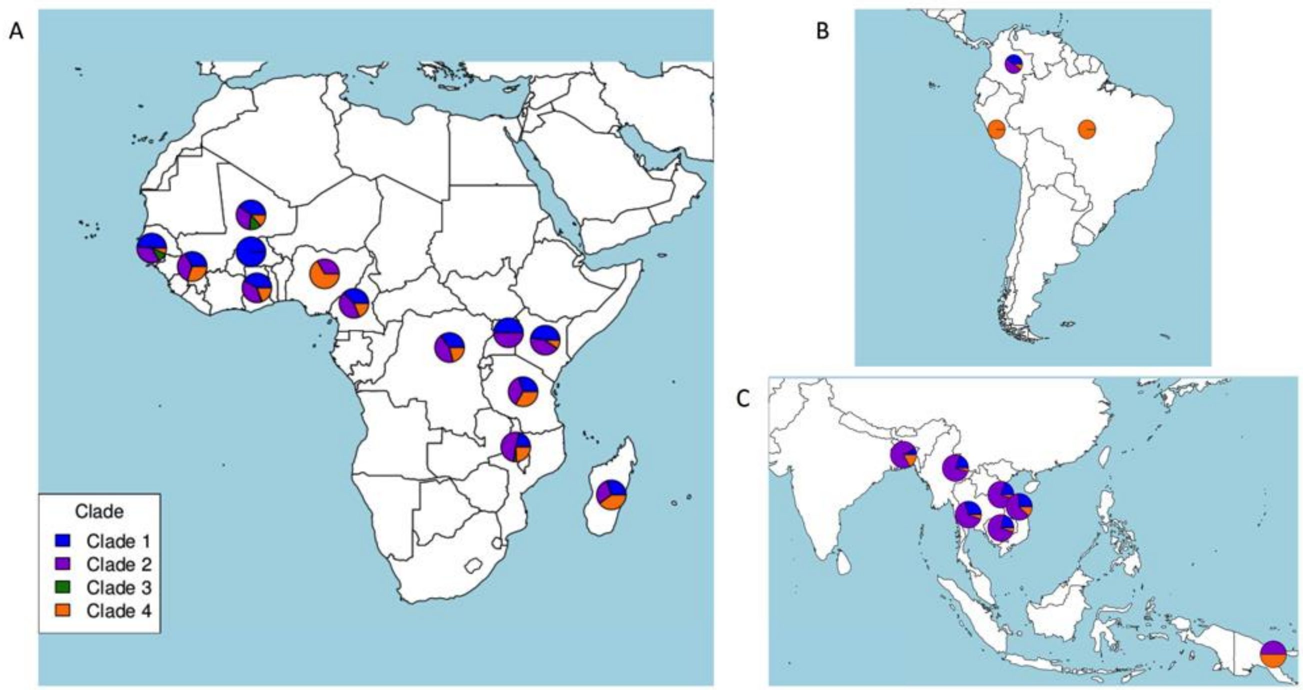

S10 figure

A rarefaction curve analysis of the haplotype diversity in the ID1-DBL2Xb region of the var2csa gene in *Plasmodium falciparum* (A; country, B; region) reveals higher diversity in African populations compared to South East Asian populations, which is supported by the neighbourhood joining trees for these regions across geography (C, D, E)

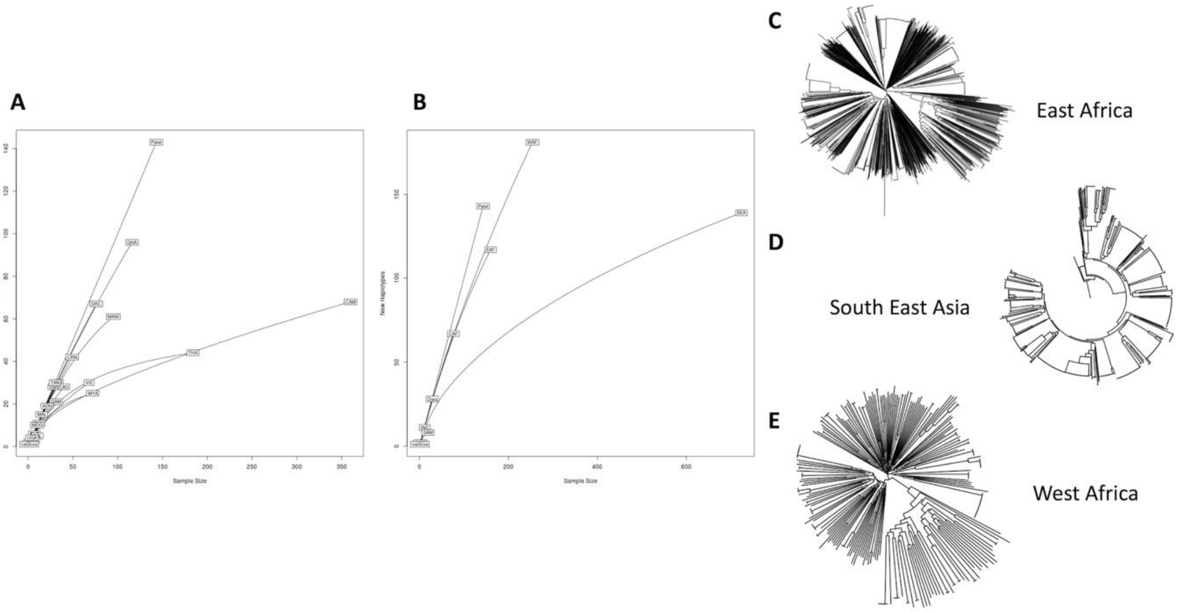

S1 table

Distribution of *P. falciparum* isolates with extra copies of the *var2csa* gene per country

	**Total**	**Single copy**		**Multiple Copies**	
**Country**	**n**	**n**	**%**	**n**	**%**
Burkina Faso	27	14	51.9	13	48.1
Cameroon	80	58	72.5	22	27.5
Gambia	47	35	74.5	12	25.5
Ghana	222	138	62.2	84	37.8
Guinea	78	51	65.4	27	34.6
Mali	29	17	58.6	12	41.4
Nigeria	6	4	66.7	2	33.3
**West Africa**	**489**	**317**	**64.8**	**172**	**35.2**
DRC	136	96	70.6	40	29.4
Kenya	26	18	69.2	8	30.8
Malawi	182	123	67.6	59	32.4
Tanzania	43	36	83.7	7	16.3
Uganda	5	4	80.0	1	20.0
Madagascar	17	12	70.6	5	29.4
**East Africa**	**409**	**289**	**70.7**	**120**	**29.3**
**Bangladesh**	**36**	**18**	**50.0**	**18**	**50.0**
Cambodia	492	428	87.0	64	13.0
Laos	88	50	56.8	38	43.2
Myanmar	109	89	81.7	20	18.3
Thailand	264	208	78.8	56	21.2
Vietnam	155	98	63.2	57	36.8
**South East Asia**	**1108**	**873**	**78.8**	**235**	**21.2**
**PNG (Oceania)**	**24**	**3**	**12.5**	**21**	**87.5**
Brazil	9	9	100.0	0	0.0
Colombia	15	14	93.3	1	6.7
Peru	9	9	100.0	0	0.0
**South America**	**33**	**32**	**97.0**	**1**	**3.0**
Overall	***2099***	***1532***	***73.0***	***567***	***27.0***

DRC Democratic Republic of Congo; PNG Papua New Guinea

S2 table

The frequency of the different protein sequence clades based on the ID1-DBL2Xb region of the *var2csa* genes in *P. falciparum*, across countries (n=1,373)

	Clade 1 (3D7-like)		Clade 2 (FCR3-like)		Clade 3		Clade 4	
**Country**	**n**	**%**	**n**	**%**	**n**	**%**	**N**	**%**
**Burkina Faso**	**7**	**100.0**	**0**	**0.0**	**0**	**0.0**	**0**	**0.0**
**Cameroon**	**7**	**100.0**	**0**	**0.0**	**0**	**0.0**	**0**	**0.0**
**Gambia**	**15**	**48.4**	**11**	**35.5**	**3**	**9.7**	**2**	**6.5**
**Ghana**	**48**	**41.7**	**44**	**38.3**	**1**	**0.9**	**22**	**19.1**
**Guinea**	**11**	**33.3**	**12**	**36.4**	**0**	**0.0**	**10**	**30.3**
**Mali**	**6**	**40.0**	**5**	**33.3**	**2**	**13.3**	**2**	**13.3**
**Nigeria**	**0**	**0.0**	**1**	**33.3**	**0**	**0.0**	**2**	**66.7**
West Africa	***94***	***44.5***	***73***	***34.6***	***6***	***2.8***	***38***	***18.0***
**DRC**	**27**	**35.5**	**33**	**43.4**	**0**	**0.0**	**16**	**21.1**
**Kenya**	**10**	**47.6**	**9**	**42.9**	**0**	**0.0**	**2**	**9.5**
**Malawi**	**20**	**21.1**	**49**	**51.6**	**3**	**3.2**	**23**	**24.2**
**Malawi***	**13**	**44.8**	**6**	**20.7**	**1**	**3.4**	**9**	**31.0**
**Malawi & Benin****	**74**	**52.1**	**59**	**41.5**	**2**	**1.4**	**7**	**4.9**
**Tanzania**	**9**	**30.0**	**11**	**36.7**	**0**	**0.0**	**10**	**33.3**
**Uganda**	**2**	**50.0**	**2**	**50.0**	**0**	**0.0**	**0**	**0.0**
**Madagascar**	**3**	**30.0**	**3**	**30.0**	**0**	**0.0**	**4**	**40.0**
East Africa	***158***	***38.8***	***172***	***42.3***	***6***	***1.5***	***71***	***17.4***
Bangladesh	1	8.3	9	75.0	0	0.0	2	16.7
Cambodia	76	21.1	266	73.9	0	0.0	18	5.0
Laos	8	20.0	30	75.0	0	0.0	2	5.0
Myanmar	14	19.4	55	76.4	0	0.0	3	4.2
Thailand	54	29.7	118	64.8	0	0.0	10	5.5
Vietnam	18	26.5	42	61.8	0	0.0	8	11.8
South East Asia	*170*	*23.5*	*511*	*70.8*	*0*	*0*	*41*	*5.7*
PNG	0	0.0	1	50.0	0	0.0	1	50.0
Brazil	0	0.0	0	0.0	0	0.0	3	100.0
Colombia	4	40.0	5	50.0	0	0.0	1	10.0
Peru	0	0.0	0	0.0	0	0.0	6	100.0
South America	*4*	*21.1*	*5*	*26.3*	*0*	*0.0*	*10*	*52.6*
Overall	*427*	*31.1*	*771*	*56.2*	*12*	*0.9*	*163*	*11.9*

*Dara et al. 2017; ** Patel el at 2017 (n=124); DRC Democratic Republic of Congo; PNG Papua New Guinea; **Bolded** denotes African Populations and Shaded denotes South East Asian Populations

S3 table

Diversity statistics by country in the open reading frame region upstream of the *var2csa* gene in 1,245 *P. falciparum* isolates

Population	n	No. of Haplotypes	Haplotype diversity (*Hd*)	Nucleotide Diversity (π)	Average no. of nucleotide differences	No. variable sites (n=360)
**Cameroon**	**46**	**28**	**0.974**	**0.020**	**7.21**	**31**
**Gambia**	**27**	**14**	**0.940**	**0.020**	**7.35**	**26**
**Ghana**	**111**	**53**	**0.973**	**0.021**	**7.42**	**38**
**Guinea**	**38**	**21**	**0.929**	**0.020**	**7.37**	**32**
**Mali**	**12**	**10**	**0.970**	**0.013**	**4.52**	**12**
**DRC**	**75**	**48**	**0.98**	**0.025**	**9.00**	**35**
**Kenya**	**16**	**12**	**0.958**	**0.017**	**6.26**	**19**
**Malawi**	**103**	**42**	**0.970**	**0.024**	**8.65**	**38**
**Tanzania**	**30**	**20**	**0.966**	**0.018**	**6.57**	**31**
**Madagascar**	**10**	**8**	**0.956**	**0.017**	**6.20**	**15**
Bangladesh	12	10	0.970	0.031	11.11	30
Cambodia	365	20	0.851	0.021	7.71	33
Laos	42	18	0.911	0.030	10.76	37
Myanmar	71	17	0.833	0.020	7.08	29
Thailand	180	30	0.934	0.028	10.05	40
Vietnam	72	23	0.867	0.025	8.89	38
Colombia	10	5	0.822	0.016	5.78	16
Overall	***1245***	***163***	***0.952***	***0.025***	***8.86***	**50**

Bolded denotes African countries and shaded denotes South East Asian countries; DRC Democratic Republic of Congo
